# Supplementary material for: Benchmarking Large Language Models for Polymer Property Predictions
Source: Macromol Rapid Commun. 2025 Oct 13;47(12):e00388. doi: 10.1002/marc.202500388 (PMC13309151; doi:10.1002/marc.202500388)
Supplement: Supplementary file 1 — Supporting File: marc70087‐sup‐0001‐SuppMat.pdf. [file MARC-47-e00388-s001.pdf]

Supporting Information for

Benchmarking Large Language Models for Polymer Property Predictions

Sonakshi Gupta,[1] Akhlak Mahmood,[2] Shivank Shukla,[2] and Rampi Ramprasad[2,*]

[1]*School of Computational Science and Engineering, Georgia Institute of Technology, 771 Ferst Drive NW, Atlanta 30332, GA, USA.*

[2]*School of Materials Science and Engineering, Georgia Institute of Technology, 771 Ferst Drive NW, Atlanta 30332, GA, USA.*

I. PROMPT DESIGN

To evaluate the influence of prompt design on model performance, five different prompt formats were explored during fine-tuning. The objective was to identify a format that minimizes RMSE and yields accurate predictions. The prompt options are outlined below:

- **Prompt 01**: *User*: "If the SMILES of a polymer is `<smiles>`, what is its `<property>`?"
  Assistant: { smiles: `<smiles>`, `<property>`: `<value><unit>` }
- **Prompt 02**: *User*: "If the SMILES of a polymer is `<smiles>`, what is its `<property>` in `<unit>`?"
  Assistant: { smiles: `<smiles>`, `<property>`: `<value>` }
- **Prompt 03**: *User*: "If the SMILES of a polymer is `<smiles>`, what is its `<property>` in `<unit>`?"
  Assistant: {`<value>`}
- **Prompt 04**: *User*: "If the SMILES of a polymer is `<smiles>`, what is its `<property>`? Answer 'not sure' if you are not certain."
  Assistant: {`<value><unit>`}
- **Prompt 05**: *User*: "If the SMILES of a polymer is `<smiles>`, what is its `<property>` in `<unit>`?"
  Assistant: { `<property>`: `<value><unit>` }

The RMSE performance of each prompt design for the T_g dataset is summarized in Figure S1.

[*] rampi.ramprasad@mse.gatech.edu

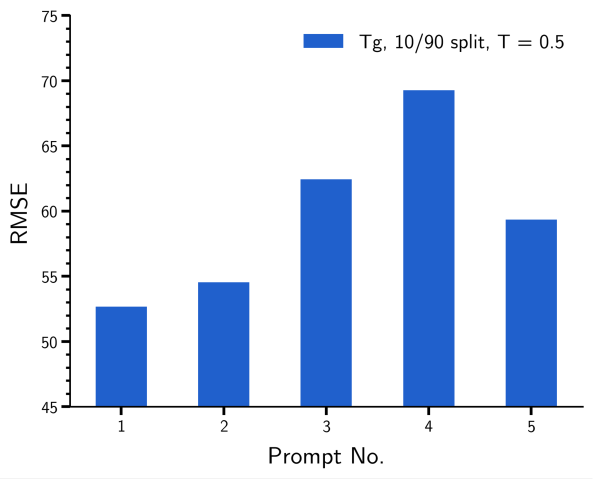

FIG. S1. Comparison of RMSE values for different prompt designs in predicting T_g, evaluated using a 10/90 train-test split with inference temperature $T = 0.5$.

II. TOKEN LENGTH DISTRIBUTIONS FOR SMILES AND INSTRUCTION-TUNED DATASETS

Token length distributions were analyzed for both the SMILES dataset and the instruction-tuned dataset across the T_g, T_m, and T_d property prediction tasks, using two tokenizers: OpenAI's `cl100k_base` and the Llama tokenizer (`meta-llama/Llama-2-7b-hf`). This analysis characterizes the typical sequence lengths encountered during model training and inference, with results presented in Figures S2 and S3.

For the SMILES dataset, the mean token length in the T_g set was 36.5 with the OpenAI tokenizer and 42.5 with the Llama tokenizer. In the T_m set, the mean lengths were 26.7 and 31.9, respectively, while in the T_d set, the values were 50.3 and 58.4. The instruction-tuned dataset, which combines the SMILES string with a natural language query, produced longer sequences. In the T_g set, the mean prompt length was 51.5 with the OpenAI tokenizer and 59.5 with the Llama tokenizer. For T_m, the mean lengths were 40.7 and 48.9, respectively, and for T_d, they were 65.3 and 75.4.

III. PARITY PLOTS AND PREDICTED VS. GROUND TRUTH DISTRIBUTIONS TO IDENTIFY CLUSTERING

The parity plots for the GPT-4o experiments are provided in Figure S4. Figure S5 presents the parity and ground truth distribution plots for Llama models fine-tuned for 5 epochs on the T_g and T_m datasets, which reveal significant clustering and poor generalization. In contrast, Figure S6 shows the ground truth distribution plots for the final optimized Llama models ($a = 16$, $r = 16$, 25 epochs) across T_g, T_m, and T_d. These plots demonstrate notable improvements in prediction diversity and distribution compared to Figure S5, justifying the enhanced performance reflected in the RMSE and BHDI metrics shown in Figure S7.

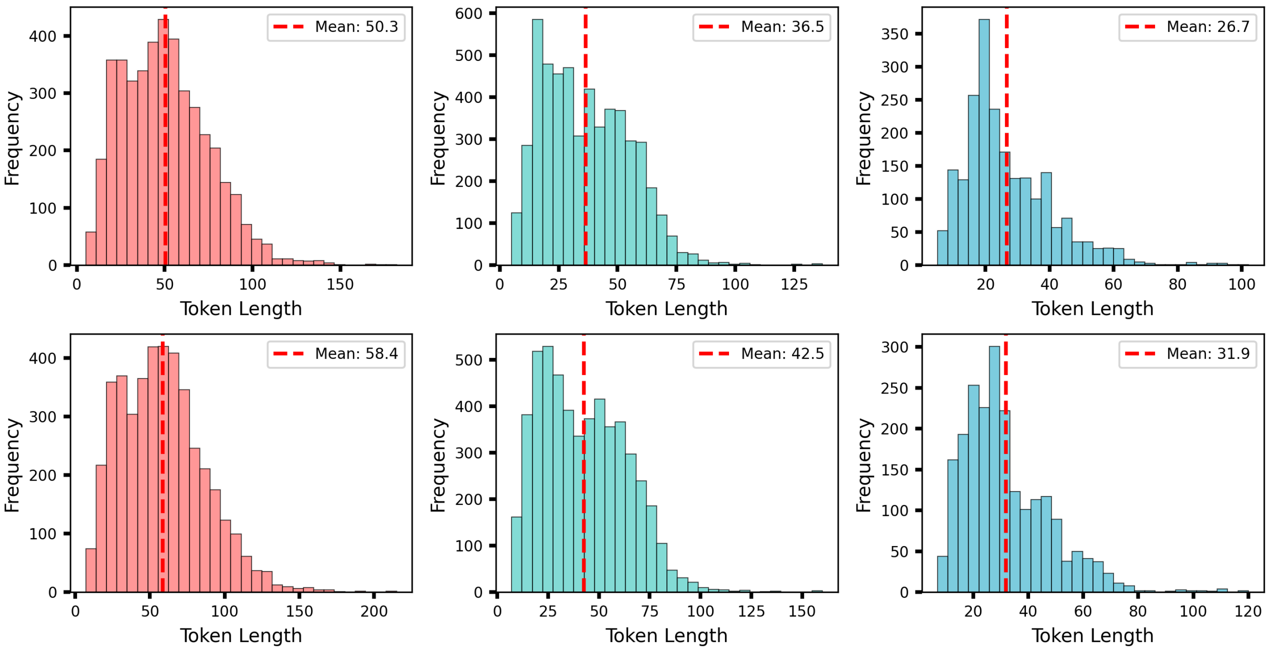

FIG. S2. Token length distributions of SMILES strings in the T_g, T_m, and T_d datasets, using the OpenAI tokenizer (top row) and Llama tokenizer (bottom row). Red dashed lines denote mean token lengths for each distribution.

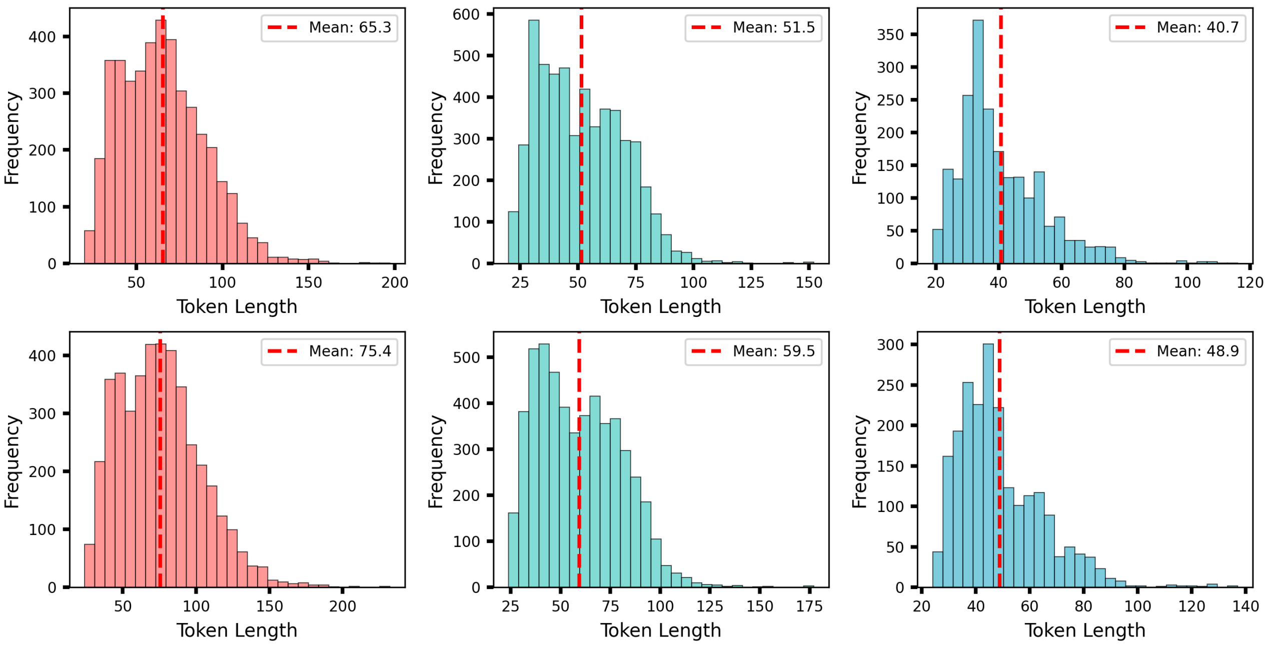

FIG. S3. Token length distributions of instruction-formatted prompts corresponding to the T_g, T_m, and T_d datasets, using the OpenAI tokenizer (top row) and Llama tokenizer (bottom row). Red dashed lines denote mean token lengths for each distribution.

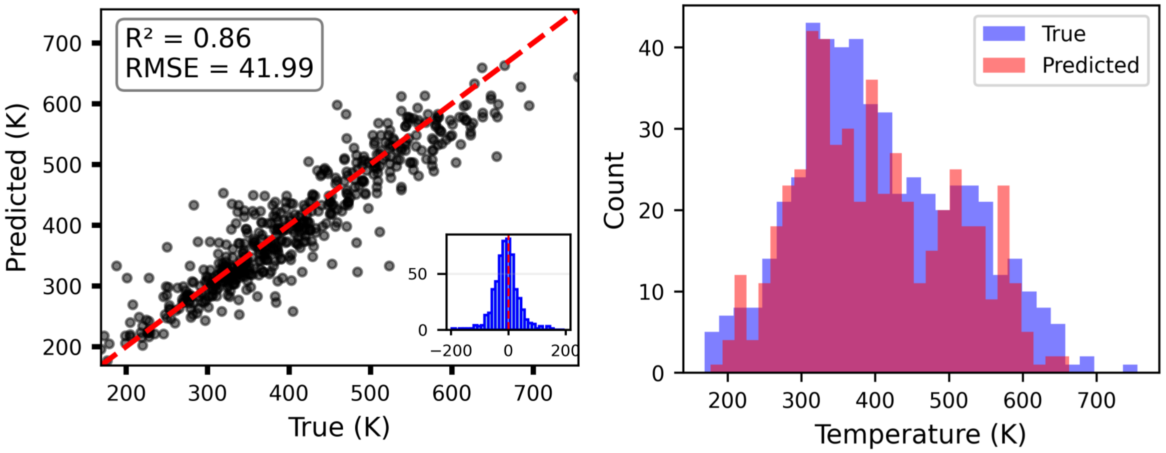

FIG. S4. Parity plot and predicted vs. ground truth data distribution plot for the fine-tuned GPT-4o model on T_g dataset.

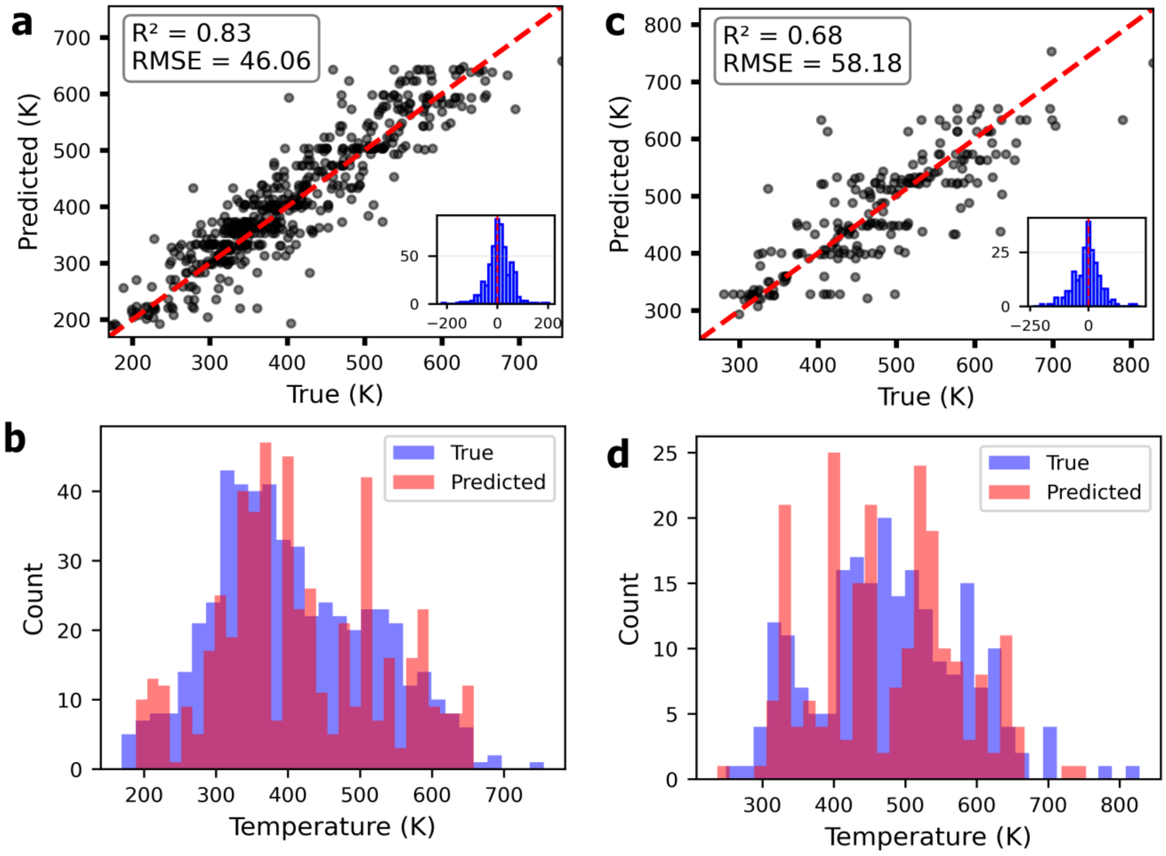

FIG. S5. Parity plot and predicted vs. ground truth data distribution plot for the fine-tuned Llama model on (a-b) T_g and (c-d) T_m datasets using configuration a= 16, r= 16 and epochs = 5. T_d was not tested due to resource constraints.

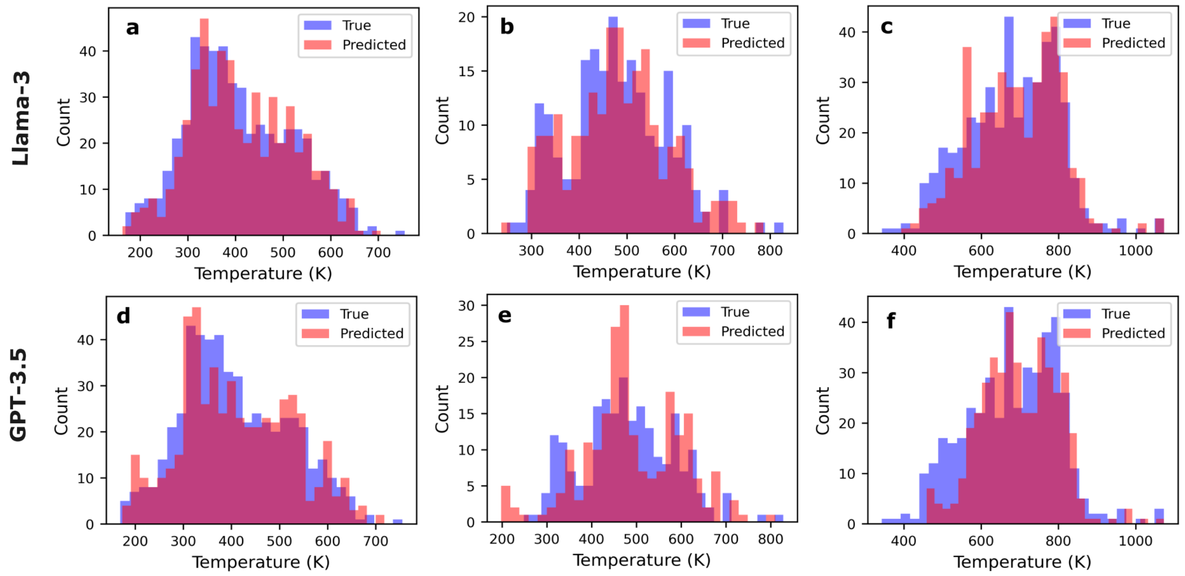

FIG. S6. Ground truth vs. predicted data distributions for T_g, T_m, T_d, respectively under ST learning: (a-c) Results for the optimized Llama model ($\alpha = 16$, $r = 16$, epochs $= 25$, $T = 0.8$) (d-f) Results for the optimized GPT fine-tuned model (epochs $= 5$, $T = 0.5$)

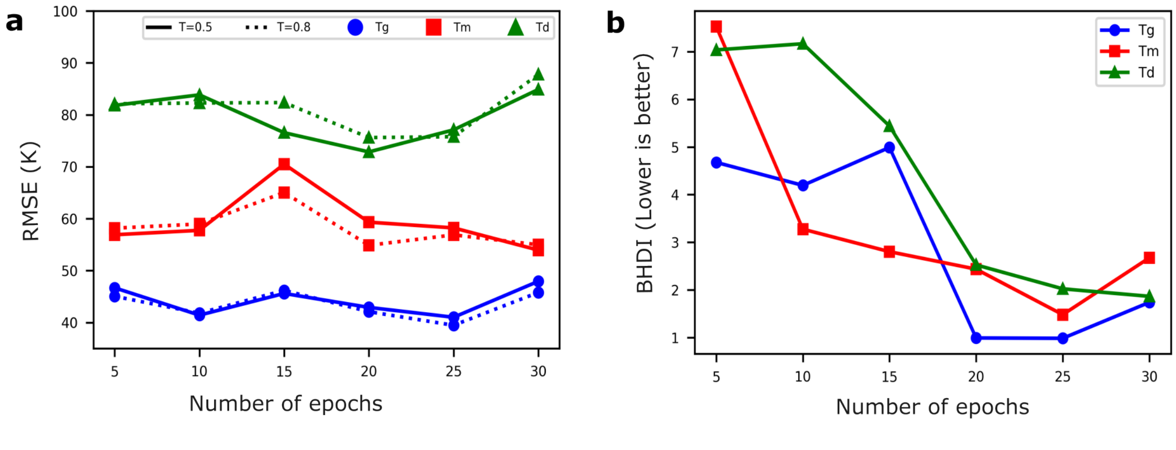

FIG. S7. Analysis done for ST learning framework. (a) RMSE trends for the fine-tuned Llama model ($\alpha = 16$, $r = 16$) across increasing epochs for three datasets (T_g, T_m, T_d), evaluated at inference temperatures $T = 0.5$ (solid lines) and $T = 0.8$ (dashed lines). (b) BHDI metric variations over epochs for the Llama model optimized at the best-performing inference temperature ($T = 0.8$).

IV. HYPERPARAMETER OPTIMIZATION OF LLMS

To optimize the performance of the fine-tuned Llama-3-8B-Instruct model, we explored various configurations of alpha (a) and rank (r) under different inference temperatures (T). The results, visualized as heatmaps in Figures S8–S12, display the RMSE values for thermal property predictions (T_g, T_m, and T_d) across a range of a and r values (16 to 128) and inference temperatures ($T = 0.5$ and $T = 0.8$) for epochs 5 and 10.

Figures S8 and S9 present the heatmaps for T_g and T_m predictions, respectively, at epochs = 5, highlighting the influence of inference temperatures ($T = 0.5$ and $T = 0.8$) on model performance. Similarly, Figures S10–S12 illustrate the heatmaps for T_g, T_m, and T_d predictions at epochs = 10, providing a detailed comparison of performance across configurations.

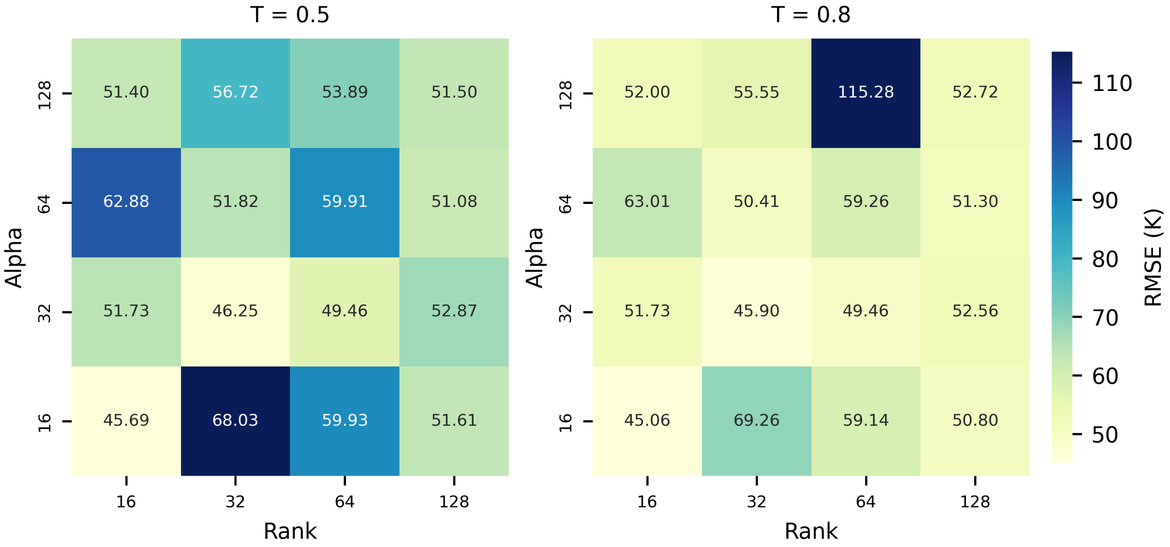

FIG. S8. Tg Prediction Heatmap for epochs = 5, inference temperature T = a) 0.5 b) 0.8

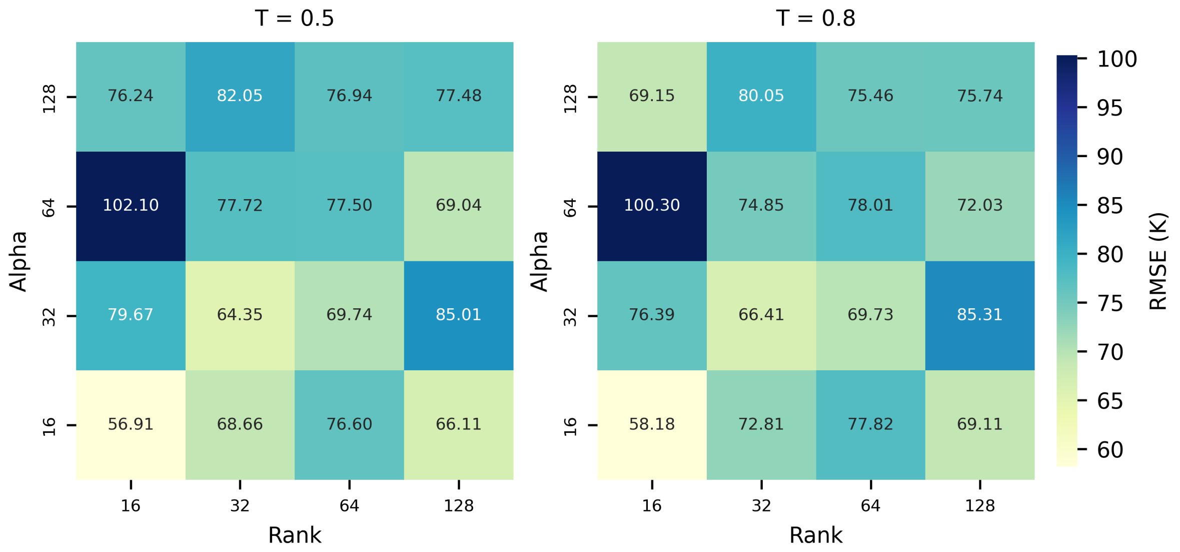

FIG. S9. Tm Prediction Heatmap for epochs = 5, inference temperature T = a) 0.5 b) 0.8

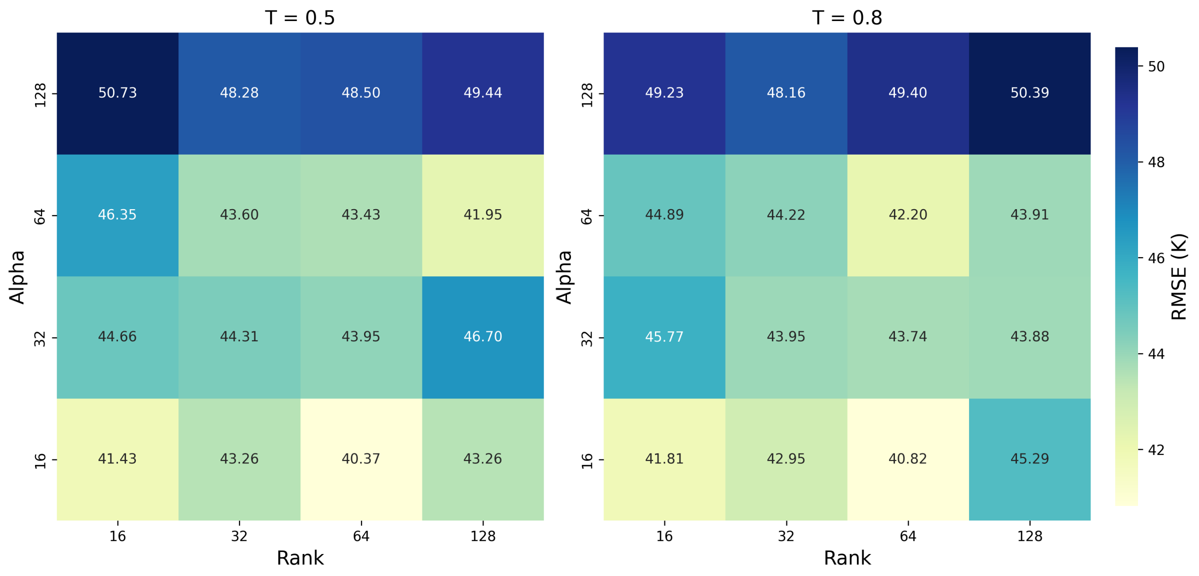

FIG. S10. Tg Prediction Heatmap for epochs = 10, inference temperature T = a) 0.5 b) 0.8

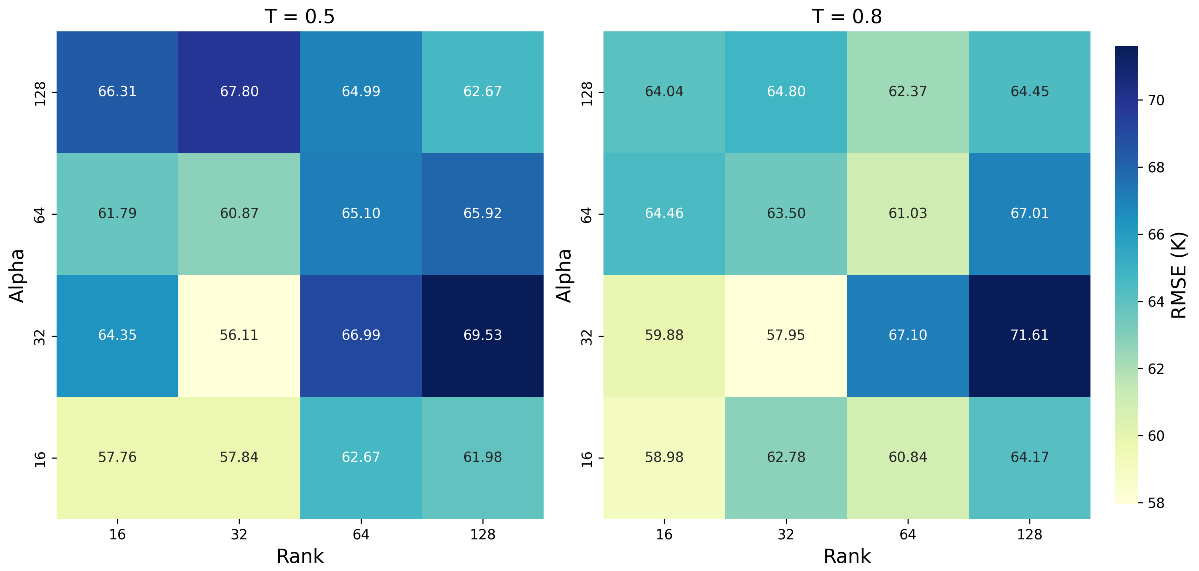

FIG. S11. Tm Prediction Heatmap for epochs = 10, inference temperature T = a) 0.5 b) 0.8

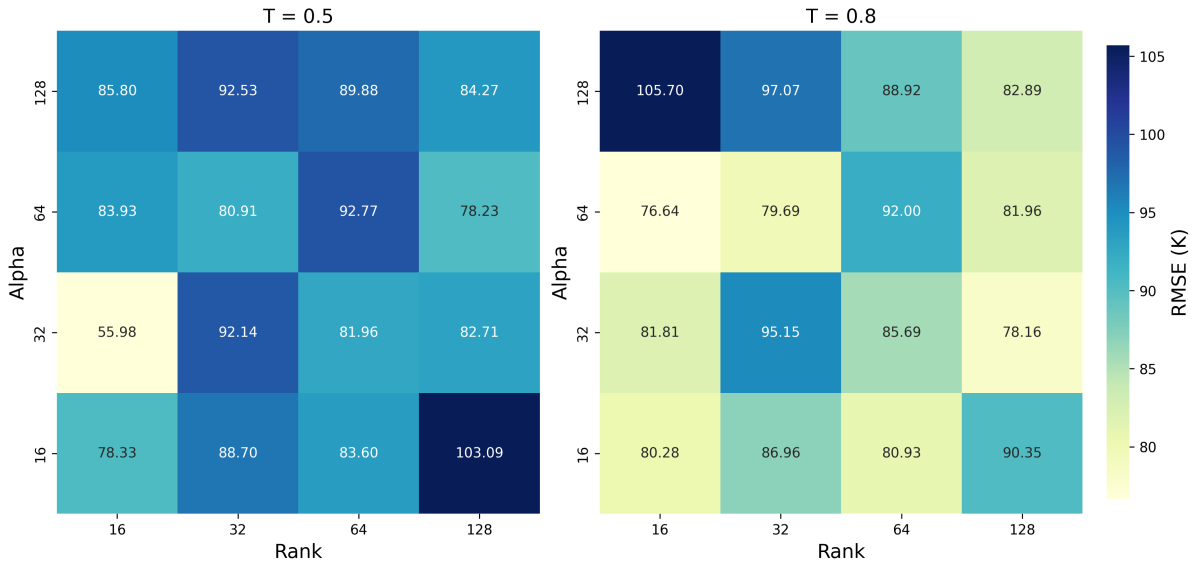

FIG. S12. Td Prediction Heatmap for epochs = 10 inference temperature T = a) 0.5 b) 0.8

V. PERFORMANCE OF DOMAIN-SPECIFIC LLM - CHEMLLM

ChemLLM [1] was fine-tuned under a single-task setup for T_g, T_m, and T_d prediction using $\alpha = 16$, rank $= 16$, and $n_{\text{epochs}} = 25$. Inference was performed at temperatures $T = 0.5$ and $T = 0.8$, with $T = 0.8$ yielding better results. These hyperparameters were selected based on the trends observed during Llama and GPT fine-tuning (Figure S5). The resulting model outputs are shown in Supplementary Figure S13. While ChemLLM captures the overall distribution reasonably well, its performance does not surpass that of Llama or GPT-based models, which consistently achieved lower RMSEs across all three properties (Figure 2-3). This indicates that further fine-tuning of a domain-specialized model does not necessarily lead to improved performance on property prediction tasks, particularly when general-purpose models are effectively optimized.

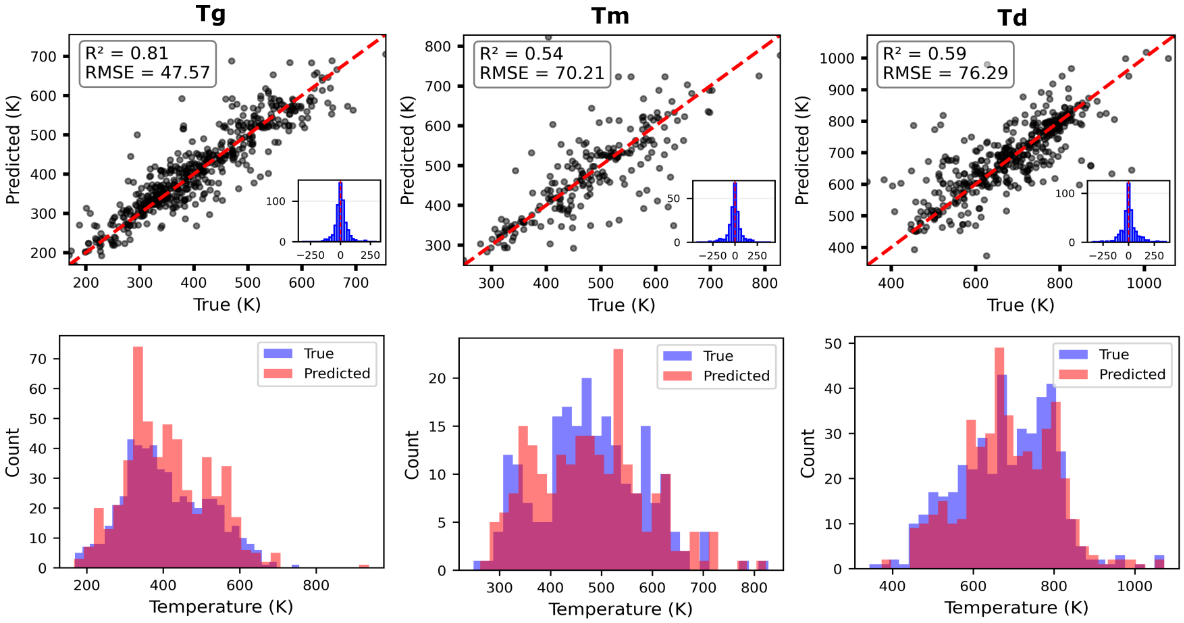

FIG. S13. Parity plot and predicted vs. ground truth data distribution plot for the fine-tuned ChemLLM model on T_g, T_m, and T_d dataset.

VI. TRAINING OF STATE-OF-THE-ART ML MODELS.

A. Polymer Genome and PolyBERT ML models

The single-task neural network and concatenation-based conditioned multitask deep neural network models, previously described by Kuenneth et al., were utilized in this study for single-task and multitask modeling of thermal properties through Polymer Genome and PolyBERT. [2] The models' inputs consisted of polymer fingerprints and selector vectors encoded via one-hot encoding to differentiate between various thermal properties, as previously established. [2]

The dataset was partitioned into training and testing subsets. Training employed a five-fold cross-validation (CV) approach, where 80% of the training dataset was used to train the CV models. The remaining 20% was used to train a meta-learner, an ensemble model constructed from the outputs of the individual CV models, to get a single prediction.

All the models were implemented through TensorFlow. [3] The Adam optimizer and Stochastic weight averaging were used to optimize the model weights. An initial learning rate of 10^{-3} was chosen and was fine-tuned using a learning rate scheduler. To prevent overfitting, we implemented early stopping and Monte Carlo dropout.

Hyperparameters, including the number of layers, neurons per layer, initial learning rate, dropout rates, and the layer for selector vector concatenation (only for multi-task model), were optimized using Hyperband in Keras Tuner. [4, 5] The same hyperparameter tuning procedure was applied to both the cross-validation and meta-learner models. The hyperparameter values for all models are listed in Tables S1 to S8.

B. PolyGNN ML models

Polymer graphs were generated from polymer SMILES strings and used as inputs to a multilayer perceptron (MLP), with the target being the thermal properties, as described in previous work by Gurnani et al. [6] The GNN models used polymer graphs as input for single-task deep neural networks, and polymer graphs combined with a selector vector (one-hot encoding different thermal properties) for multitask models. The model architecture consisted of five-fold cross-validation models without a meta-learner. All models were implemented using PyTorch. [7] Model optimization and weight updates were implemented through the Adam optimizer, stochastic weight averaging, and learning rate scheduler. Hyperparameter optimization included tuning the number of layers and the number of neurons per layer, with detailed hyperparameter specifications provided in Tables S9–S12.

TABLE S1. Best Hyperparameters for CV + Metalearner Polymer Genome Single task Tg

	CV					Meta
	Fold 0	Fold 1	Fold 2	Fold 3	Fold 4	Meta
Selector vector concatenated at layer	-	-	-	-	-	-
Number of hidden layers	3	3	3	3	3	2
Nodes in the first hidden layer	352	480	480	416	544	320
Nodes in the second hidden layer	480	416	480	416	416	448
Nodes in the third hidden layer	480	544	544	416	480	-
Epoch	300	300	300	300	300	300

TABLE S2. Best Hyperparameters for CV + Metalearner Polymer Genome Single task Tm

	CV					Meta
	Fold 0	Fold 1	Fold 2	Fold 3	Fold 4	Meta
Selector vector concatenated at layer	-	-	-	-	-	-
Number of hidden layers	3	3	3	3	3	2
Nodes in the first hidden layer	352	480	480	416	544	256
Nodes in the second hidden layer	480	416	480	416	416	256
Nodes in the third hidden layer	480	544	544	416	480	-
Epoch	300	300	300	300	300	300

TABLE S3. Best Hyperparameters for CV + Metalearner Polymer Genome Single task Td

	CV					Meta
	Fold 0	Fold 1	Fold 2	Fold 3	Fold 4	Meta
Selector vector concatenated at layer	-	-	-	-	-	-
Number of hidden layers	3	3	3	3	3	2
Nodes in the first hidden layer	416	416	416	480	480	448
Nodes in the second hidden layer	480	480	352	544	416	512
Nodes in the third hidden layer	480	480	352	480	480	-
Epoch	300	300	300	300	300	300

TABLE S4. Best Hyperparameters for CV + Meta learner Polymer Genome Multi task

	CV					Meta
	Fold 0	Fold 1	Fold 2	Fold 3	Fold 4	Meta
Selector vector concatenated at layer	0	0	2	2	2	-
Number of hidden layers	3	3	3	3	3	2
Nodes in the first hidden layer	416	416	480	416	480	384
Nodes in the second hidden layer	480	480	480	352	352	384
Nodes in the third hidden layer	416	416	480	544	480	-
Epoch	300	300	300	300	300	300

TABLE S5. Best Hyperparameters for CV + Metalearner PolyBERT Single task Tg

	CV					Meta
	Fold 0	Fold 1	Fold 2	Fold 3	Fold 4	Meta
Selector vector concatenated at layer	-	-	-	-	-	-
Number of hidden layers	3	3	3	3	3	2
Nodes in the first hidden layer	416	480	544	544	544	320
Nodes in the second hidden layer	480	416	480	480	544	448
Nodes in the third hidden layer	416	480	416	416	544	-
Epoch	300	300	300	300	300	300

TABLE S6. Best Hyperparameters for CV + Metalearner PolyBERT Single task Tm

	CV					Meta
	Fold 0	Fold 1	Fold 2	Fold 3	Fold 4	Meta
Selector vector concatenated at layer	-	-	-	-	-	-
Number of hidden layers	3	3	3	3	3	2
Nodes in the first hidden layer	480	416	480	480	480	448
Nodes in the second hidden layer	416	480	416	416	416	384
Nodes in the third hidden layer	544	416	416	544	480	-
Epoch	300	300	300	300	300	300

TABLE S7. Best Hyperparameters for CV + Metalearner PolyBERT Single task Td

	CV					Meta
	Fold 0	Fold 1	Fold 2	Fold 3	Fold 4	Meta
Selector vector concatenated at layer	-	-	-	-	-	-
Number of hidden layers	3	3	3	3	3	2
Nodes in the first hidden layer	352	416	352	416	480	512
Nodes in the second hidden layer	480	480	480	352	416	448
Nodes in the third hidden layer	480	416	480	416	480	-
Epoch	300	300	300	300	300	300

TABLE S8. Best Hyperparameters for CV + Metalearner PolyBERT Multi task

	CV					Meta
	Fold 0	Fold 1	Fold 2	Fold 3	Fold 4	Meta
Selector vector concatenated at layer	2	2	2	2	2	-
Number of hidden layers	3	3	3	3	3	2
Nodes in the first hidden layer	480	544	544	480	544	512
Nodes in the second hidden layer	352	416	544	352	480	320
Nodes in the third hidden layer	480	352	480	480	416	-
Epoch	300	300	300	300	300	300

TABLE S9. Best Hyperparameters for CV PolyGNN Single task Tg

	Fold 0	Fold 1	Fold 2	Fold 3	Fold 4	Meta
Selector vector concatenated at layer	-	-	-	-	-	-
Number of hidden layers	5	5	5	5	5	-
Nodes in the first hidden layer	64	64	64	64	64	-
Nodes in the second hidden layer	64	64	64	64	64	-
Nodes in the third hidden layer	64	64	64	64	64	-
Nodes in the fourth hidden layer	64	64	64	64	64	-
Nodes in the fifth hidden layer	32	32	32	32	32	-
Epoch	300	300	300	300	300	-

TABLE S10. Best Hyperparameters for CV PolyGNN Single task Tm

	Fold 0	Fold 1	Fold 2	Fold 3	Fold 4	Meta
Selector vector concatenated at layer	-	-	-	-	-	-
Number of hidden layers	3	3	3	3	3	-
Nodes in the first hidden layer	64	64	64	64	64	-
Nodes in the second hidden layer	64	64	64	64	64	-
Nodes in the third hidden layer	32	32	32	32	32	-
Epoch	1000	1000	1000	1000	1000	-

TABLE S11. Best Hyperparameters for CV PolyGNN Single task Td

	Fold 0	Fold 1	Fold 2	Fold 3	Fold 4	Meta
Selector vector concatenated at layer	-	-	-	-	-	-
Number of hidden layers	6	6	6	6	6	-
Nodes in the first hidden layer	64	64	64	64	64	-
Nodes in the second hidden layer	64	64	64	64	64	-
Nodes in the third hidden layer	64	64	64	64	64	-
Nodes in the fourth hidden layer	64	64	64	64	64	-
Nodes in the fifth hidden layer	64	64	64	64	64	-
Nodes in the sixth hidden layer	32	32	32	32	32	-
Epoch	1000	1000	1000	1000	1000	-

TABLE S12. Best Hyperparameters for CV PolyGNN Multi task

	Fold 0	Fold 1	Fold 2	Fold 3	Fold 4	Meta
Selector vector concatenated at layer	-	-	-	-	-	-
Number of hidden layers	3	3	3	3	3	-
Nodes in the first hidden layer	128	128	128	128	128	-
Nodes in the second hidden layer	64	64	64	64	64	-
Nodes in the third hidden layer	32	32	32	32	32	-
Epoch	100	100	100	100	100	-

SUPPLEMENTARY REFERENCES

[1] D. Zhang, W. Liu, Q. Tan, J. Chen, H. Yan, Y. Yan, J. Li, W. Huang, X. Yue, W. Ouyang, D. Zhou, S. Zhang, M. Su, H.-S. Zhong, and Y. Li, Chemllm: A chemical large language model (2024), arXiv:2402.06852 [cs.AI].

[2] C. Kuenneth, A. C. Rajan, H. Tran, L. Chen, C. Kim, and R. Ramprasad, Polymer informatics with multi-task learning, Patterns **2**, 100238 (2021).

[3] M. Abadi, A. Agarwal, P. Barham, E. Brevdo, Z. Chen, C. Citro, G. Corrado, A. Davis, J. Dean, M. Devin, S. Ghemawat, I. Goodfellow, A. Harp, G. Irving, M. Isard, Y. Jia, R. Jozefowicz, L. Kaiser, M. Kudlur, J. Levenberg, D. Mane, R. Monga, S. Moore, D. Murray, C. Olah, M. Schuster, J. Shlens, B. Steiner, I. Sutskever, P. Talwar, P. Tucker, V. Vanhoucke, V. Vasudevan, F. Viegas, O. Vinyals, P. Warden, M. Wattenberg, M. Wicke, Y. Yu, and X. Zheng, Tensorflow: Large-scale machine learning on heterogeneous systems, arXiv:1603.04467 (2016), arXiv:1603.04467 [cs.DC].

[4] L. Li, K. Jamieson, G. DeSalvo, A. Rostamizadeh, and A. Talwalkar, Hyperband: A novel bandit-based approach to hyperparameter optimization, in *International Conference on Learning Representations (ICLR)* (2017).

[5] T. O'Malley and E. Bursztein, Keras tuner, `https://github.com/keras-team/keras-tuner` (2019).

[6] R. Gurnani, C. Kuenneth, A. Toland, and R. Ramprasad, Polymer informatics at scale with multitask graph neural networks, Chemistry of Materials **35**, 1560 (2023).

[7] A. Paszke, S. Gross, F. Massa, A. Lerer, J. Bradbury, G. Chanan, T. Killeen, Z. Lin, N. Gimelshein, L. Antiga, *et al.*, Pytorch: An imperative style, high-performance deep learning library, in *Advances in Neural Information Processing Systems* (2019) pp. 8024–8035.
